# Beyond Accuracy: A Reliability-Oriented Multi-Dimensional Benchmark of Data-Driven Fault Diagnosis for Liquid Rocket Engines

**DOI:** 10.3390/s26185923

**Published:** 2026-09-19

**Authors:** Mingyang Geng, Gang Zheng, Long He, Siyu Zhao, Xiuwei Yu

**Affiliations:** Xichang Satellite Launch Center, Xichang 615000, China; gengmingyang13@nudt.edu.cn (M.G.); 18882898793@163.com (L.H.); rryu49453@126.com (S.Z.); yuxiuwei1991@163.com (X.Y.)

**Keywords:** liquid rocket engine, fault diagnosis, machine learning, deep learning, feature importance

## Abstract

Liquid rocket engines (LREs) are mission-critical propulsion systems whose reliability directly affects launch safety and the repeated operation of reusable launch vehicles. Data-driven LRE fault diagnosis remains difficult because labeled fault samples are scarce, engine-to-engine variations cause distribution shifts, and existing evaluations frequently emphasize classification accuracy without controlling for input representation. This study establishes a reliability-oriented benchmark that evaluates seven representative machine-learning and deep-learning methods from four dimensions: diagnostic capability, sensor informativeness, cross-unit generalization, and data efficiency. The experiments use 1106 fault samples from the XJTU-REF dataset, covering nine fault categories, 19 sensor channels, and three simulated engine units. To ensure an apples-to-apples model comparison, all seven methods are first evaluated using the same 19-dimensional steady-state representation. Under this controlled setting, Random Forest achieves the highest accuracy of 89.15% and Macro-F1 score of 88.74%, while the best deep model, 1D-CNN, reaches 86.42%. An additional representation-ablation experiment shows that concatenating the same steady-state features with learned sequence representations increases 1D-CNN accuracy to 87.58%, reducing its gap from Random Forest to 1.57 percentage points. This result demonstrates that the large difference observed under the original heterogeneous-input setting is partly attributable to representation design rather than model family alone. In cross-unit evaluation, Random Forest obtains an average accuracy of 75.37%, whereas Transformer obtains 61.54%, indicating that engine-to-engine variation remains a major deployment challenge. The top five sensors contribute 47.62% of the total Random Forest importance, and, with five labeled samples per fault category, KNN and Random Forest achieve 71.24% and 70.82% accuracy, respectively. These findings provide quantitative guidance for algorithm selection, sensor prioritization, and data-acquisition planning in reusable launch vehicle health-monitoring systems.

## 1. Introduction

Liquid rocket engines (LREs) are among the most mission-critical and failure-sensitive subsystems of modern launch vehicles. They operate under extreme temperature, pressure, vibration, and propellant-flow conditions, while their thrust and mixture-control characteristics directly determine vehicle trajectory and mission completion. Historical events illustrate this system-level criticality. During the STS-51F mission, a premature shutdown of one Space Shuttle Main Engine forced an abort-to-orbit trajectory, and the first Ariane 5 ECA flight was lost after a failure associated with the Vulcain 2 engine nozzle cooling system [1,2]. For reusable launch vehicles, propulsion reliability is even more demanding because an engine must retain acceptable performance and fault tolerance over repeated operating cycles. Accurate, robust, and transferable fault diagnosis is therefore a fundamental requirement for next-generation aerospace health-monitoring systems.

Traditional LRE fault-diagnosis approaches can be grouped into three major paradigms: model-based methods, signal-processing-based methods, and data-driven intelligent methods. Model-based approaches use physical models, parameter estimation, state observers, and residual analysis to identify deviations from nominal engine behavior [3,4,5,6]. They offer strong physical interpretability, but their reliability depends on model fidelity and accurate characterization of complex nonlinear engine dynamics. Signal-processing-based methods extract fault-sensitive information through principal component analysis, frequency-domain analysis, dynamic time warping, and time–frequency Transforms [7,8,9,10]. These methods reduce dependence on a complete physical model but often require expert-designed features and may be sensitive to noise and operating-condition variation. More recently, machine-learning and deep-learning approaches have demonstrated the ability to learn nonlinear diagnostic relationships from multi-sensor data [11,12,13,14,15,16,17].

Despite this progress, practical deployment remains constrained by three coupled problems. First, real LRE fault samples are rare because engines are designed for high reliability, and destructive fault experiments are costly and risky. Second, a model trained on one engine unit may degrade on another unit because of manufacturing tolerance, component variation, and operating differences. Third, conclusions regarding model superiority can be confounded by the input representation: a classical classifier supplied with a curated steady-state vector is not directly comparable with a deep network that must learn both temporal representation and classification from the full sequence using the same limited number of labeled samples. Consequently, a useful benchmark must control input information while separately studying the value of temporal representation learning.

Existing studies on the XJTU-REF dataset and related LRE diagnosis tasks have generally focused on validating individual algorithms. Four limitations remain insufficiently addressed: (1) model families are rarely compared under an identical input representation; (2) sensor contribution and measurement redundancy are not jointly evaluated with diagnostic accuracy; (3) cross-unit transfer across simulated engine realizations is seldom examined for all benchmarked methods; and (4) the relationship between the number of labeled samples and diagnostic performance is not quantified systematically. These limitations constitute the scientific and engineering gap addressed in this work.

**Figure 1:**
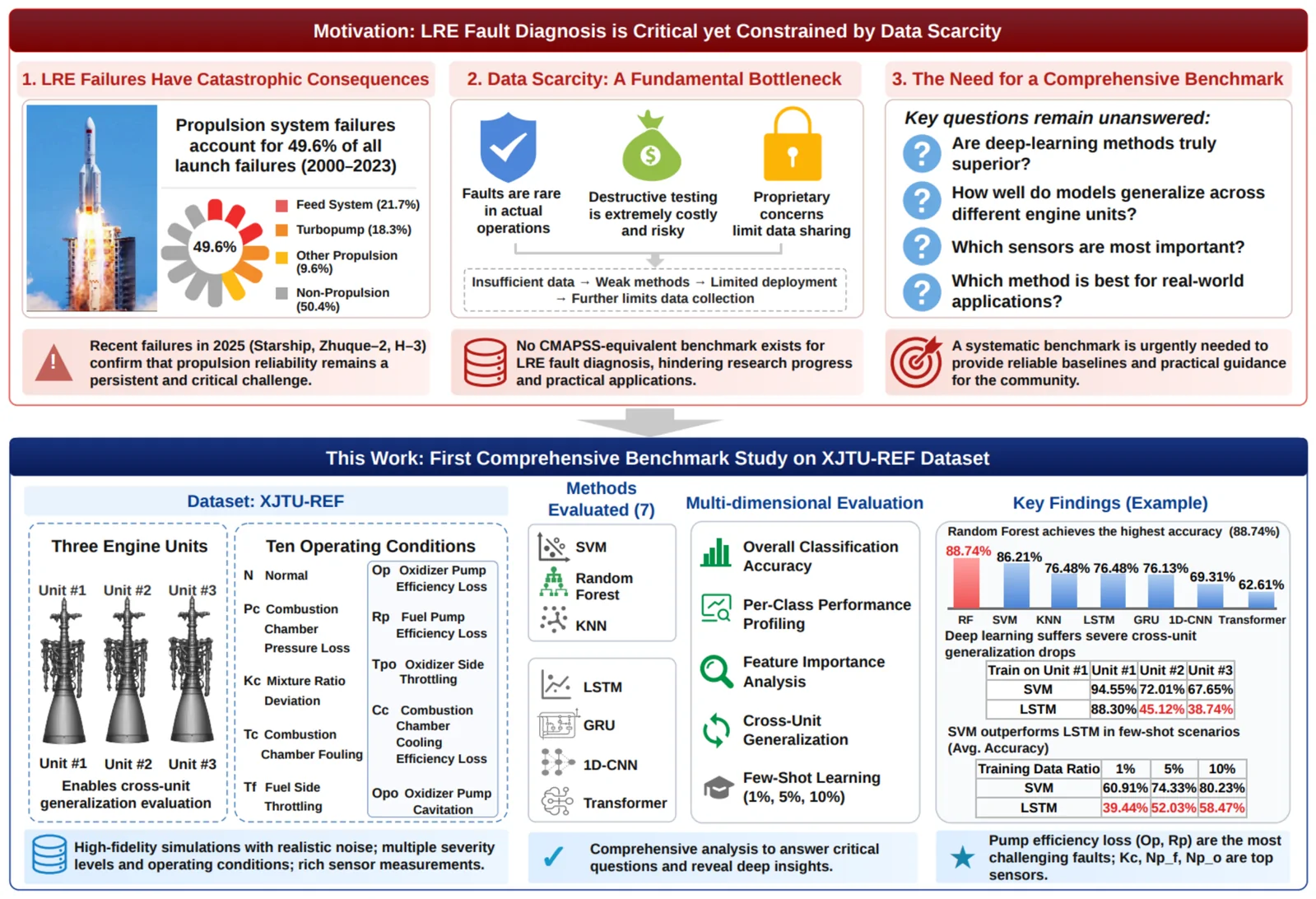
Motivation and structure of the reliability-oriented benchmark. The need for reliable LRE diagnosis arises from propulsion-system criticality, scarce labeled fault data, engine-to-engine variation, and incomplete evaluation protocols. These challenges are addressed through controlled diagnostic comparison, sensor-informativeness analysis, cross-unit evaluation, and few-shot evaluation.

As illustrated in Figure 1, the logical structure of this study proceeds from the engineering criticality of LREs to three practical constraints—limited fault data, engine-to-engine variation, and incomplete evaluation protocols—and then to four corresponding evaluation dimensions. The text and the subsequent experimental sections follow the same order as the figure. The objective of this work is not to introduce another fault classifier, but to determine how model architecture, input representation, sensor information, engine-unit variation, and data availability jointly affect diagnostic reliability. The study addresses the following research questions:

RQ1: When all methods receive the same diagnostic information, how do classical machine-learning and deep-learning models compare, and how much of the observed performance difference is attributable to input representation?
RQ2: Which sensor measurements provide the greatest contribution to fault discrimination?
RQ3: How robust are the seven diagnostic methods when transferred to an unseen simulated engine unit?
RQ4: How efficiently do the seven methods learn when only a small number of labeled samples are available for each fault category?

The principal contributions are summarized as follows:

- **A representation-controlled benchmark is established.** All seven methods are first evaluated using the same steady-state sensor vector, and a separate sequence/steady-state fusion experiment quantifies the effect of supplying deep models with the same hand-crafted information. This design separates model-family effects from representation effects.
- **Reliability is evaluated beyond aggregate accuracy.** The benchmark jointly considers overall and class-level performance, a complete normalized confusion matrix, sensor importance, cross-unit transfer, few-shot learning, model variance, and computational cost.
- **All seven methods are retained in the deployment-oriented experiments.** SVM, Random Forest, KNN, LSTM, GRU, 1D-CNN, and Transformer are evaluated in both cross-unit and few-shot settings, avoiding conclusions based on only one classical and one deep model.
- **Quantitative engineering guidance is derived.** The results identify the strongest model under controlled limited-data conditions, quantify the reduction caused by engine-unit shift, identify dominant sensors, and estimate the labeled-data requirements associated with different method families.

The remainder of this paper is organized as follows. Section 2 reviews the principal LRE fault-diagnosis paradigms and summarizes their unresolved limitations. Section 3 presents the dataset, preprocessing, representation-controlled comparison, sequence/steady-state fusion, and the four experimental protocols. Section 4 reports the results in the same order: controlled diagnostic performance and error analysis, sensor informativeness, cross-unit generalization, and few-shot capability. Section 5 discusses the roles of representation, model complexity, fault similarity, unit shift, and sensor configuration. Section 6 summarizes the principal quantitative findings and future directions.

## 2. Related Work

### 2.1. Fault-Diagnosis Methods for Liquid Rocket Engines

Fault diagnosis for liquid rocket engines has evolved through three primary paradigms over the past decades: model-based methods, signal-processing-based methods, and data-driven intelligent methods. Each paradigm emerged in response to specific technological contexts and application demands, progressively shifting from physical interpretability toward adaptability and automation.

Model-based methods represent the earliest systematic approach to LRE fault diagnosis. These methods leverage mathematical physical models of the engine to estimate parameters and detect faults through residual analysis; the estimated parameters are compared with measured parameters, and residual deviations indicate anomalies [3]. This paradigm offers clear physical interpretability and provides a theoretical foundation for fault localization. Representative works include Maru et al. [3], who combined autoregressive moving-average models with extended Kalman filters to develop a residual-based method using Mahalanobis distance for fault detection. Similarly, Kanso et al. [4] employed extended Kalman filtering to estimate combustion chamber health status, supporting remaining useful life prediction. However, the diagnostic performance of model-based methods heavily relies on the accuracy of the engine model, and establishing precise physical models for complex LRE systems is often challenging in practice. Moreover, the generalizability of such models is relatively poor, limiting their applicability across different engine configurations or operating conditions [5,6].

As model-based methods faced practical limitations, signal-processing techniques emerged as an alternative data-driven approach. These methods extract fault features from measurement signals using techniques such as principal component analysis (PCA), Fast Fourier Transform (FFT), and time–frequency analysis, bypassing the need for explicit physical models. Tsutsumi et al. [7] used PCA combined with dynamic time warping (DTW) to achieve LRE fault detection on a two-dimensional phase plane. Traditional techniques including frequency analysis, parity-space methods, and wavelet denoising were also widely applied during this period [8,9]. Compared to model-based approaches, signal-processing methods offer greater flexibility and reduced dependence on system-specific physics. Nevertheless, these methods are inherently limited by noise sensitivity, the requirement for strong signal correlations, and heavy reliance on expert knowledge for feature engineering, features that may not generalize across different engine types, operating conditions, or fault modes [10].

With the advent of artificial intelligence and the growing availability of computational resources, data-driven intelligent methods have gained increasing attention. Unlike previous paradigms, these methods do not rely on precise physical models or extensive expert knowledge but instead directly leverage observable data from the engine system, demonstrating strong adaptive and learning capabilities. Conventional machine-learning models, including support vector machines (SVMs), artificial neural networks (ANNs), and Random Forests, combined with hand-crafted features have been successfully applied to turbopump and steady-state fault detection [11,12,13,18,19,20]. More recently, deep-learning architectures such as convolutional neural networks (CNNs), long short-term memory networks (LSTMs), and autoencoders have enabled end-to-end feature learning and classification, eliminating the need for manual feature engineering [14,15,16,17]. For instance, Xing et al. [21] constructed fault datasets using numerical simulations and achieved multi-category fault identification based on deep-learning models. Flora et al. [22] designed an ANN-based algorithm for sensor fault isolation and replacement, thereby enhancing system fault tolerance.

A critical bottleneck for data-driven methods in LRE applications is the scarcity of real fault samples, as engines are designed for high reliability, and fault data are extremely rare in practice. To address this challenge, generative adversarial networks (GANs), introduced by Goodfellow et al., have emerged as powerful data-generation tools. Through adversarial training between generator and discriminator, GANs can synthesize samples that closely match the distribution of real data, offering a novel pathway to augment limited fault datasets. Recent research specifically targeting LREs has demonstrated the effectiveness of integrating temporal modeling with generative frameworks. Deng et al. [10] proposed an FDD method for large LOX/kerosene rocket engines based on an LSTM-GAN architecture, which uses LSTM to capture the dynamic characteristics of the engine’s steady-state and startup processes. Cheng et al. [23] proposed a GAN framework based on Wasserstein distance, enabling high-precision LRE fault detection by simulating the distribution of normal data. Despite these advancements, instability during GAN training remains a key issue that affects the quality of generated data, and enhancing the authenticity, diversity, and controllability of the generated data remains a significant focus of current research.

The evolution of LRE fault-diagnosis methods reflects a clear trajectory: from physics-driven residual analysis (model-based), to expert-dependent feature extraction (signal processing), and toward fully data-adaptive learning (intelligent data-driven). Each paradigm has distinct strengths and limitations. Model-based methods excel in interpretability but suffer from model accuracy dependency; signal-processing methods offer flexibility but require expert knowledge; data-driven intelligent methods provide superior adaptability and automation but face challenges in data scarcity, model interpretability, and training stability. Future research is likely to focus on hybrid approaches that integrate physical knowledge with data-driven learning, as well as advanced generative techniques and transfer learning strategies to further alleviate the data bottleneck and improve diagnostic robustness across diverse operational scenarios.

To clarify the unresolved limitations of existing approaches, Table 1 compares the major fault-diagnosis paradigms used in LRE applications. Each paradigm is effective under particular assumptions, but no single paradigm resolves the combined problems of limited data, representation dependence, engine-to-engine variation, and sensor-system design.

The comparison shows that the principal research gap is not the absence of diagnostic algorithms, but the absence of a unified evaluation protocol that controls input information and simultaneously considers fault-level performance, sensor contribution, cross-unit robustness, and data efficiency. This gap motivates the benchmark developed in this study.

### 2.2. The XJTU-REF Dataset

The XJTU-REF dataset, developed by researchers at Xi’an Jiaotong University, addresses the critical gap in LRE fault data availability through high-fidelity simulation [24]. The dataset was constructed using a component-level decomposition approach, where each subsystem, pump, valve, turbine, pipe, cavitation tube, gas generator, and combustion chamber was modeled separately, and then integrated into a complete dynamic system simulation using MATLAB/Simulink (R2023b). The simulation incorporates 19 sensor signals sampled at 500 Hz across ten operating conditions, including one normal condition and nine fault types: valve faults (oxidizer main valve, fuel main valve), combustion chamber faults (oxidizer injector clogging, combustion chamber ablation), gas generator line faults (oxygen secondary line clogging, gas generator fuel leakage), and turbopump faults (oxidizer-pump-efficiency loss, fuel-pump-efficiency loss, turbine gas leakage). The dataset incorporates three “engine units” per fault type, with slight variations in cavitation coefficients to simulate manufacturing tolerances and operational differences between engines.

### 2.3. Research Gap and Motivation

Existing LRE diagnosis studies have established useful model-based, signal-processing, machine-learning, and deep-learning solutions, but their results are difficult to compare because the evaluated datasets, input representations, and deployment assumptions differ. In particular, the literature does not yet provide a unified benchmark that (1) controls diagnostic information across model families, (2) reports complete fault-level error patterns, (3) evaluates all benchmarked methods across engine units, and (4) quantifies their data efficiency under identical few-shot splits.

This study addresses these gaps through a representation-controlled benchmark of seven methods and a separate temporal-feature-fusion ablation. The framework evaluates diagnostic capability, sensor informativeness, cross-unit robustness, and data efficiency using consistent partitions and metrics.

## 3. Methodology

### 3.1. Overview of the Reliability-Oriented Benchmark Framework

The benchmark is implemented through four sequential stages. First, the XJTU-REF samples are organized according to fault category and simulated engine unit, after which sensor normalization and input-representation construction are performed using training data only. Second, all seven diagnostic methods are evaluated under a representation-controlled protocol. Third, sequence-only and sequence/steady-state fusion experiments are conducted for the four deep models to quantify representation effects. Fourth, sensor informativeness, cross-unit generalization, and few-shot performance are evaluated. The ordering of the methodology subsections is identical to that of Section 4, allowing every experimental procedure to be associated directly with its corresponding result.

### 3.2. Dataset Description and Preprocessing

#### 3.2.1. Dataset Composition

The XJTU-REF dataset is selected because it provides synchronized multi-sensor sequences, multiple component-level fault categories, and explicit simulated engine-unit identities. As summarized in Table 2, the available data contain 1106 fault samples distributed among nine fault categories and three samples representing healthy operation. Each sample contains d=19 sensor channels and T=501 time steps.

Only three healthy samples are available in the dataset version used in this study. Because this number is insufficient for statistically meaningful five-fold stratification, the primary benchmark is formulated as a nine-class fault-type classification task. The healthy samples are retained in Table 2 for a complete description of the dataset, but they are not included in the quantitative cross-validation metrics, per-class analysis, confusion matrix, cross-unit evaluation, or few-shot evaluation. Accordingly, the number of evaluated classes is C=9.

The dataset supports the study objectives in four ways. The 1106 fault samples permit controlled comparison across nine fault types; the 19 synchronized channels support sensor-contribution analysis; the three simulated engine units support source-to-target transfer evaluation; and at least 114 samples are available for every fault category, permitting few-shot support sets of up to 20 samples per class while retaining independent test samples. Nevertheless, the dataset remains simulation-based and represents only a limited range of unit variation; the results should therefore be interpreted as a controlled benchmark rather than conclusive evidence of fleet-level reliability.

#### 3.2.2. Data Preprocessing

Let the *i*-th sample be represented as $X_{i}=\left[x_{itj}\right]\in R^{T\times d}$, where *i* is the sample index, t∈{1,…,T} is the time-step index, j∈{1,…,d} is the sensor index, T=501, and d=19. Each sensor channel is standardized according to

(1) $$x_{itj}^{\prime }=\frac{x_{itj}-\mu_{j}}{\sigma_{j}},$$

where $x_{itj}$ and $x_{itj}^{\prime }$ denote the original and standardized measurements, respectively, and $\mu_{j}$ and $\sigma_{j}$ are the mean and standard deviation of sensor *j* estimated exclusively from the training subset.

The final 20% of each standardized sequence is used to construct the steady-state representation. Let

(2) $$T_{s}=\left\lceil 0.8T\right\rceil$$

be the first time index of the steady-state interval. The steady-state value of sensor *j* in sample *i* is

(3) $$x_{ij}^{\mathrm{steady}}=\frac{1}{T-T_{s}+1}\sum_{t=T_{s}}^{T}x_{itj}^{\prime },$$

where $T-T_{s}+1$ is the number of time points included in the average. The complete steady-state vector is

(4) $$x_{i}^{\mathrm{steady}}={\left[x_{i1}^{\mathrm{steady}},\ldots ,x_{id}^{\mathrm{steady}}\right]}^{T}\in R^{d}.$$

All normalization parameters and derived representations are calculated without using test samples. In cross-unit experiments, $\mu_{j}$ and $\sigma_{j}$ are estimated from the source unit only; in few-shot experiments, they are estimated from the support set only.

#### 3.2.3. Task Definition and Class-Imbalance Treatment

The main task is nine-class fault classification. Moderate imbalance remains among the fault classes because their sample counts range from 114 to 147. Therefore, accuracy is reported together with Macro-F1 and balanced accuracy. All repeated within-unit evaluations use stratification based on the nine fault labels. The same cross-validation folds are used for every method.

### 3.3. Evaluated Diagnostic Methods and Fair-Comparison Protocol

#### 3.3.1. Classical Machine-Learning Methods

Three classical methods are evaluated: support vector machine (SVM), Random Forest (RF), and K-Nearest Neighbor (KNN). SVM uses a linear kernel and a one-versus-one multi-class strategy. RF combines bootstrap sampling and random feature selection across multiple decision trees. KNN provides a non-parametric distance-based baseline. In every experiment, these methods receive the standardized 19-dimensional steady-state vector $x_{i}^{\mathrm{steady}}$.

#### 3.3.2. Deep-Learning Methods

Four deep architectures are evaluated: long short-term memory (LSTM), Gated Recurrent Unit (GRU), one-dimensional convolutional neural network (1D-CNN), and Transformer. LSTM and GRU model gated dependencies, 1D-CNN learns local interactions, and Transformer uses self-attention to model global relationships.

#### 3.3.3. Representation-Controlled Comparison

The primary benchmark controls the diagnostic information supplied to every method. All seven methods receive the same 19 standardized steady-state values. For the four deep models, the vector is represented as a sequence of 19 sensor tokens, one token per sensor channel. A learnable sensor-identity embedding is added to each token so that the network can distinguish sensor channels. No original temporal sample is available in this controlled setting. Consequently, differences in the primary benchmark reflect model behavior on identical input information rather than differences between hand-crafted and automatically learned temporal representations.

#### 3.3.4. Sequence and Feature-Fusion Ablation

To evaluate the effect of temporal representation, the deep models are additionally trained under two auxiliary settings:

(a) **Sequence only:** the standardized sequence $X_{i}^{\prime }\in R^{501\times 19}$ is supplied directly to the deep model.
(b) **Sequence–steady-state fusion:** the hidden representation $h_{i}^{\mathrm{seq}}$ learned from the full sequence is concatenated with the same steady-state vector used by the classical methods:
  (5) $$z_{i}=\left[h_{i}^{\mathrm{seq}}\;∥\;x_{i}^{\mathrm{steady}}\right],$$
  where ∥ denotes vector concatenation. The fused representation $z_{i}$ is passed to the final classification layer.

The representation-controlled experiment is used for the principal comparison among model families. The sequence-only and fusion experiments are interpreted as representation ablations and are not used to support a blanket claim that one model family is universally superior. Our whole reliability-oriented benchmark protocol is illustrated in Algorithm 1.

### 3.4. Experimental Design

#### 3.4.1. RQ1: Controlled Diagnostic Performance and Error Analysis

Five-fold stratified cross-validation is conducted, and the same folds are used for all seven methods. The primary outcomes are accuracy, Macro-F1, balanced accuracy, training cost, per-class recall, and a row-normalized confusion matrix. The representation ablation is evaluated on exactly the same partitions.

#### 3.4.2. RQ2: Sensor-Informativeness Analysis

Random Forest importance is calculated because RF provides the strongest controlled baseline while supporting direct feature-contribution estimation. For sensor *j*, the unnormalized importance is

(6) $${\tilde{I}}_{j}=\frac{1}{N_{T}}\sum_{k=1}^{N_{T}}\Delta G_{k}\left(j\right),$$

and the normalized importance is

(7) $$I_{j}=\frac{{\tilde{I}}_{j}}{\sum_{\ell =1}^{d}{\tilde{I}}_{\ell }},$$

where $N_{T}$ is the number of trees, $\Delta G_{k}\left(j\right)$ is the cumulative weighted reduction in Gini impurity generated by splits using sensor *j* in tree *k*, d=19, ${\tilde{I}}_{j}$ is the unnormalized importance, and $I_{j}$ is the normalized importance satisfying $\sum_{j=1}^{d}I_{j}=1$. The percentage reported in the results is $100I_{j}\%$.
**Algorithm 1** Reliability-Oriented Benchmark Protocol**Require:** Nine-class fault dataset D; methods M; engine-unit labels; repeated random seeds
**Ensure:** Multi-dimensional benchmark results
  1:Split D into training and test subsets using the prescribed protocol  2:Fit sensor normalization using training data only  3:Construct $x^{\mathrm{steady}}$ for every training and test sample  4:**for** each method m∈M **do**  5:   Train and evaluate *m* using the common steady-state representation  6:   Record accuracy, Macro-F1, balanced accuracy, variance, and cost  7:**end for**  8:**for** each deep model **do**  9:   Evaluate the sequence-only representation10:    Evaluate sequence–steady-state fusion using Equation (5)11:**end for**12:Compute per-class recall and the complete normalized confusion matrix13:Estimate and rank sensor importance using the RF model14:**for** each ordered source–target engine-unit pair **do**15:    Train all seven methods on the source unit using the controlled representation16:    Test all seven methods on the unseen target unit17:**end for**18:**for **N∈{1,2,3,5,10,20}**do**19:    Sample *N* labeled examples from each of the nine fault classes20:    Train and evaluate all seven methods using the same support/test split21:**end for**22:Aggregate the repeated results and derive engineering implications **return** Benchmark report


To further examine whether the feature-importance ranking can translate into an actual reduction in sensor channels, a sensor-subset ablation experiment is conducted using Random Forest. The sensors are ranked according to their feature importance calculated from the training data, and the classifier is retrained using only the top-*k* ranked sensors:

(8) k∈{3,5,8,10,19}.

The k=19 configuration corresponds to the complete sensor set and is used as the reference baseline. For each repeated evaluation, sensor ranking is determined using the training subset only, and the selected sensor subset is then applied to the corresponding test subset to avoid information leakage.

This ablation is designed to evaluate whether the information identified by the feature-importance analysis is sufficiently concentrated to preserve diagnostic performance with fewer measurements. It should be noted that feature importance is used only to rank candidate sensor subsets and is not interpreted as direct evidence that lower-ranked sensors are redundant.

#### 3.4.3. RQ3: Cross-Unit Generalization Evaluation

An engine unit denotes a complete simulated realization of the same nominal LRE architecture with unit-specific component parameters. It does not denote an individual sample, fault category, or time point. Let $u_{s}$ and $u_{t}$ denote different source and target units. The cross-unit setting is

(9) $$P_{\mathrm{train}}\left(X,Y\right)=P^{\left(u_{s}\right)}\left(X,Y\right)\neq P^{\left(u_{t}\right)}\left(X,Y\right)=P_{\mathrm{test}}\left(X,Y\right),$$

where X is the input representation, *Y* is the fault label, and $P^{\left(u_{s}\right)}$ and $P^{\left(u_{t}\right)}$ are the source- and target-unit joint distributions. All six ordered transfer directions are evaluated: Unit 1 → Unit 2, Unit 1 → Unit 3, Unit 2 → Unit 1, Unit 2 → Unit 3, Unit 3 → Unit 1, and Unit 3 → Unit 2. No target-unit sample is used for scaling, training, hyperparameter selection, or early stopping. All seven methods use the common steady-state representation in this experiment.

#### 3.4.4. RQ4: Few-Shot Diagnostic Capability

Let *N* denote the number of labeled training samples selected independently from each fault category:

(10) N∈{1,2,3,5,10,20}.

The support set contains 9N samples, and the remaining samples form the test set. The sampling is repeated five times using fixed random seeds. The healthy samples are excluded because they cannot provide independent support and test observations. All seven methods use the same support set, test set, and controlled steady-state representation for each repetition.

### 3.5. Evaluation Metrics

Accuracy is defined as

(11) $$\mathrm{Accuracy}=\frac{N_{\mathrm{correct}}}{N_{\mathrm{total}}},$$

where $N_{\mathrm{correct}}$ is the number of correctly classified test samples and $N_{\mathrm{total}}$ is the total number of test samples. For category *c*,

$${\mathrm{Precision}}_{c}=\frac{TP_{c}}{TP_{c}+FP_{c}},\;{\mathrm{Recall}}_{c}=\frac{TP_{c}}{TP_{c}+FN_{c}},$$

where $TP_{c}$, $FP_{c}$, and $FN_{c}$ are the true-positive, false-positive, and false-negative counts. The class-specific F1 score is

$$F1_{c}=\frac{2\;{\mathrm{Precision}}_{c}\;{\mathrm{Recall}}_{c}}{{\mathrm{Precision}}_{c}+{\mathrm{Recall}}_{c}}.$$

Macro-F1 and balanced accuracy are

(12) $$Macro\mathrm{-F}1=\frac{1}{C}\sum_{c=1}^{C}F1_{c},$$

(13) $$\mathrm{Balanced}\;\mathrm{Accuracy}=\frac{1}{C}\sum_{c=1}^{C}{\mathrm{Recall}}_{c},$$

where C=9. Mean and standard deviation are reported across repeated evaluations.

### 3.6. Implementation Details

All experiments are implemented using Python 3.9, TensorFlow 2.13, and scikit-learn 1.3. Hyperparameters for the classical methods are selected by grid search using training data only. Deep models use Adam optimization, a batch size of 32, a maximum of 80 epochs, and early stopping with a patience of 15 epochs. Fixed random seeds are used for all repeated experiments. Test samples and target-unit samples are never used during preprocessing, hyperparameter selection, or early stopping. The controlled, sequence-only, and fusion experiments use identical train/test partitions.

## 4. Experimental Results

### 4.1. RQ1: Controlled Diagnostic Performance and Error Analysis

#### 4.1.1. Representation-Controlled Comparison

Table 3 reports the primary comparison in which all seven methods receive exactly the same 19-dimensional steady-state information.

RF achieves the highest controlled accuracy of 89.15±1.22%, followed by KNN at 88.31±1.37%. The best deep architecture is 1D-CNN at 86.42±2.11%. The RF–1D-CNN difference is therefore 2.73 percentage points, which is substantially smaller than the difference obtained when classical models receive steady-state features and deep models receive unprocessed full sequences. The controlled comparison supports a narrower conclusion: RF provides the best combination of accuracy and stability for this dataset, but the results do not support a general claim that classical methods are intrinsically superior to deep learning.

The deep methods exhibit larger standard deviations and higher training costs. For example, RF has a standard deviation of 1.22 percentage points, whereas Transformer has a standard deviation of 3.01 points. This indicates greater sensitivity of the higher-capacity models to the limited training sample.

#### 4.1.2. Effect of Input Representation

Table 4 separates model architecture from representation design for the four deep models.

The sequence-only setting produces the lowest performance for every deep architecture. When the same steady-state information used by the classical methods is concatenated with the learned sequence representation, accuracy increases by 10.28–23.56 percentage points. The fused 1D-CNN reaches 87.58%, only 1.57 points below RF. Therefore, the large gap in the original heterogeneous-input comparison was partly caused by the requirement that deep models learn both temporal-feature extraction and classification from approximately one thousand labeled samples. The fusion results also show that temporal models can remain competitive when physically meaningful summary information is made explicitly available.

#### 4.1.3. Per-Class Performance

The difficult categories are model-dependent. As shown in Table 5, under RF, Ojet and Ov obtain recalls of 56.7% and 61.7%, substantially below the other categories. In contrast, RF recognizes the two pump-efficiency faults Op and Rp with recalls of 95.8% and 96.7%. Therefore, the revised results do not support the original general statement that pump-efficiency degradation is always the most difficult diagnostic problem. A more defensible conclusion is that faults affecting neighboring portions of the oxidizer-delivery path are strongly confusable, whereas pump-fault difficulty depends on the selected representation and classifier.

#### 4.1.4. Complete Confusion Analysis

Table 6 reports the complete row-normalized confusion matrix for RF rather than only selected pairs.

For readability, Table 7 lists the largest off-diagonal entries ranked over all class pairs.

The dominant confusion is bidirectional between Ojet and Ov. Both faults affect the oxidizer-delivery path and can produce related changes in flow, mixture ratio, and chamber conditions. The complete matrix also demonstrates that Ofu does not dominate every misclassification; the previous table appeared Ofu-centered because only selected pairs were reported. Reporting the full matrix removes this ambiguity and avoids the appearance of cherry-picking. Although these faults originate at different physical locations, both affect the oxidizer-delivery path and can effectively restrict oxidizer supply to the combustion chamber. As a result, they may produce similar steady-state variations in oxidizer flow, mixture-ratio-related measurements, and coupled combustion responses.

### 4.2. RQ2: Sensor-Informativeness Analysis

As shown in Table 8, the top five sensors account for 47.62% of the total RF importance. Combustion mixture ratio $K_{c}$ ranks first at 12.09%, followed by the fuel- and oxidizer-pump power measurements $N_{pf}$ and $N_{po}$. The ranking indicates that global combustion balance and turbopump behavior provide complementary diagnostic information. However, feature importance should not be interpreted as evidence that the remaining channels can be removed without validation; correlated sensors may share importance, and sensor-subset performance should be verified in a separate ablation study before hardware reduction.

Table 9 evaluates whether the feature-importance ranking can support a reduced sensor configuration. When only the Top-3 sensors are retained, the diagnostic accuracy decreases to 82.93%, indicating that the three highest-ranked measurements alone do not contain sufficient information for robust multi-fault discrimination.

When the sensor subset is expanded to the Top-5 measurements, the accuracy increases to 87.62% with a Macro-F1 score of 87.08%. Compared with the complete 19-sensor configuration, which achieves 89.15% accuracy, the Top-5 subset introduces an absolute accuracy reduction of only 1.53 percentage points while using approximately 26.3% of the original sensor channels.

Further increasing the number of sensors to eight and ten gradually improves the accuracy to 88.54% and 88.91%, respectively. The complete 19-sensor configuration still provides the highest overall performance. These results indicate that the diagnostic information is concentrated in a relatively small number of high-ranked measurements, while lower-ranked sensors provide complementary information that contributes to the final diagnostic performance.

Therefore, the sensor-importance ranking should be interpreted as a basis for identifying candidate reduced sensor configurations rather than as direct evidence that low-ranked sensors can be removed without performance loss.

### 4.3. RQ3: Cross-Unit Generalization Performance

Table 10 includes all seven methods and all six ordered transfer directions. The common steady-state representation is used throughout so that differences reflect model robustness rather than unequal input information.

RF provides the highest mean cross-unit accuracy of 75.37% and the smallest mean degradation of 13.78 percentage points. Among the deep models, 1D-CNN performs best with a mean of 68.08%, but its decrease of 18.34 points remains larger than that of RF, KNN, and SVM. Transformer shows the largest mean degradation, decreasing from 83.68% within unit to 61.54% across units. These findings show that the cross-unit gap persists even after input representation is controlled, although the degradation is less extreme than in the original sequence-only LSTM comparison.

### 4.4. RQ4: Few-Shot Diagnostic Capability

As shown in Table 11, KNN provides the highest reference performance from one to ten samples per fault category, whereas RF becomes strongest at 20 samples per class and in the full-data condition. With five samples per category, KNN and RF achieve 71.24% and 70.82%, compared with 60.48% for 1D-CNN and 52.76% for LSTM. At one shot, KNN reaches 45.12%, while Transformer reaches 15.83%. The results indicate that local and ensemble decision rules can exploit the curated steady-state representation more efficiently under extreme sample scarcity. As the support set grows, the performance difference decreases, particularly for 1D-CNN.

### 4.5. Summary of Statistical Reliability

The controlled benchmark shows that model stability is an independent consideration from average accuracy. RF combines the highest mean accuracy with the smallest reported standard deviation, whereas the deep models generally exhibit larger variation across partitions. The representation-ablation results further show that variance decreases when the steady-state vector is supplied explicitly to the deep model. Accordingly, the revised conclusions are based jointly on mean performance, variability, representation control, and deployment-oriented evaluations rather than on a single accuracy value.

## 5. Discussion

### 5.1. Input Representation Is a Major Part of the Apparent Model Gap

The revised experiments change the interpretation of the classical-versus-deep-learning comparison. Under the original heterogeneous-input setting, classical methods received an averaged steady-state vector, whereas deep models were required to extract useful temporal features from 501-step sequences and perform classification using approximately one thousand labeled samples. That comparison combined two effects: model architecture and representation burden.

When the input information is controlled, RF remains the strongest method at 89.15%, but 1D-CNN reaches 86.42%, reducing the difference to 2.73 points. When the same steady-state vector is fused with the learned temporal representation, 1D-CNN reaches 87.58%, only 1.57 points below RF. Therefore, the appropriate conclusion is not that classical methods universally outperform deep models. Rather, RF offers the most favorable accuracy–stability–cost trade-off for the present limited dataset, while deep models become competitive when physically informative summary features are made explicitly available.

This result has a practical implication for benchmark design: model families should not be ranked without controlling the information supplied to them. It also suggests a useful engineering direction in which domain-informed steady-state features and learned temporal representations are combined instead of treated as mutually exclusive alternatives.

### 5.2. Fault Difficulty Is Architecture- and Path-Dependent

The complete confusion matrix identifies Ojet and Ov as the dominant confusion pair for RF. Both faults affect the oxidizer-delivery path, and their effects can propagate to overlapping flow, mixture-ratio, and chamber-response patterns. In contrast, the pump-efficiency faults Op and Rp are recognized reliably by RF but remain more difficult for some deep architectures. Fault difficulty should therefore be reported by model and representation rather than summarized through a single universal ranking.

This finding also illustrates why the complete matrix is necessary. A selected-pair table can overemphasize one fault code and may create the impression that the analysis was chosen after observing the results. Reporting all matrix entries and then ranking the largest off-diagonal values provides a transparent basis for the physical interpretation.

### 5.3. Cross-Unit Generalization Remains a Deployment Bottleneck

All methods deteriorate when the source and target engine units differ. RF decreases from 89.15% within a unit to a six-direction mean of 75.37%, while Transformer decreases from 83.68% to 61.54%. Because the same steady-state representation is used for every method, this difference cannot be attributed solely to unequal input information. The results indicate that higher-capacity models remain more sensitive to unit-specific relationships under the current data scale.

Nevertheless, the revised cross-unit findings are less extreme than the original comparison between SVM and a sequence-only LSTM. This distinction is important: sequence-only degradation quantifies the combined effect of representation learning and domain shift, whereas the controlled experiment more directly quantifies model robustness to unit shift. Future work should evaluate domain adaptation, invariant-risk learning, physics-guided normalization, and multi-unit pretraining using the same controlled protocol.

### 5.4. Sensor Importance Supports Prioritization but Not Immediate Removal

The top five measurements contribute 47.62% of RF impurity importance, with $K_{c}$, $N_{pf}$, and $N_{po}$ occupying the first three positions. The ranking supports prioritization of combustion-balance and turbopump measurements during sensor-system design. However, importance is not equivalent to dispensability. Correlated sensors can divide or exchange importance, and low-ranked measurements may remain essential for a particular rare fault. Sensor removal should therefore be justified through subset retraining, fault-specific recall analysis, and robustness testing under sensor noise or failure.

### 5.5. Data Availability Should Determine the Initial Deployment Strategy

The few-shot experiment shows that KNN and RF provide strong performance when only one to ten labeled samples are available per fault category. With five samples per class, KNN reaches 71.24%, compared with 60.48% for 1D-CNN and 52.76% for LSTM. These results favor lightweight or instance-based models for an initial deployment in which labeled fault data are extremely limited. As more multi-unit data become available, fused deep models may become preferable because they can exploit both explicit steady-state information and transient sequence patterns.

Accordingly, method selection should be staged rather than fixed. A classical baseline can provide transparent and data-efficient initial capability, while a fusion model can be introduced after sufficient representative data have been accumulated and its cross-unit behavior has been independently validated.

### 5.6. Physical Interpretation of the Ojet–Ov Confusion

The confusion matrix shows that Ojet and Ov form the most prominent bidirectional confusion pair. This behavior can be physically interpreted from their common influence on the oxidizer-delivery path. Ojet represents a restriction near the oxidizer injector, whereas Ov represents abnormal flow regulation at the oxidizer main valve. Although the fault locations are different, both mechanisms can reduce the effective oxidizer supply and therefore produce similar downstream changes in oxidizer flow, mixture ratio, and combustion-related measurements.

The controlled benchmark further uses steady-state features obtained by averaging the final 20% of each signal sequence. This representation suppresses part of the transient response and emphasizes the final operating state. Consequently, fault mechanisms that originate at different physical locations but lead to similar steady-state oxidizer-flow restrictions can become difficult to distinguish in the feature space.

This result suggests that separating Ojet and Ov may require measurements that contain stronger localization information, such as local pressure differences or transient responses around the oxidizer valve and injector. Therefore, combining steady-state global indicators with localized or transient measurements may improve fault isolation in future LRE diagnostic systems.

### 5.7. Implications for Sensor Configuration and Health Monitoring

The sensor-importance analysis identifies combustion mixture ratio ($K_{c}$), turbopump-related measurements ($N_{pf}$ and $N_{po}$), and major flow measurements as the most informative diagnostic variables. However, feature importance should not be interpreted directly as sensor redundancy.

The sensor-subset ablation provides additional evidence for this issue. The Top-5 sensor configuration achieves 87.62% accuracy, compared with 89.15% using all 19 measurements, corresponding to a reduction of only 1.53 percentage points while retaining approximately 98.3% of the full-sensor accuracy. Nevertheless, the complete sensor set still achieves the highest performance, indicating that lower-ranked measurements contain complementary diagnostic information. Therefore, sensor ranking can support candidate sensor-reduction strategies, but practical sensor removal should also consider fault coverage, redundancy, reliability, hardware complexity, and safety requirements.

### 5.8. Limitations and Future Directions

This study has five principal limitations. First, the numerical benchmark is based on a simulation dataset; sensor noise, unmodeled dynamics, environmental variation, and maintenance history may differ from flight data. Second, only three simulated engine units are available, limiting the diversity of cross-unit evaluation. Third, the controlled deep-model input treats sensor channels as tokens and therefore evaluates nonlinear sensor interaction rather than temporal learning; the sequence/fusion ablation is required to interpret temporal capability. Fourth, RF impurity importance can be affected by feature correlation and should be complemented by permutation importance, SHAP analysis, and physical sensitivity analysis. Fifth, the healthy condition contains only three samples and cannot support a statistically defensible normal-versus-fault study. Future datasets should contain substantially more healthy runs, more engine units, multiple fault severities, and repeated operating cycles. Future benchmarks should also include early detection, open-set recognition, sensor-failure robustness, domain adaptation, and simulation-to-real validation.

## 6. Conclusions

This study established a representation-controlled, reliability-oriented benchmark for data-driven LRE fault diagnosis. The revised protocol excludes the three healthy samples from quantitative nine-class evaluation, supplies the same steady-state information to all seven methods for the primary comparison, and evaluates all seven methods in cross-unit and few-shot settings. Under the controlled representation, RF achieves the highest reference accuracy of 89.15% and Macro-F1 of 88.74%, while 1D-CNN achieves 86.42%. Supplying the same steady-state features to the temporal branch through feature fusion increases 1D-CNN accuracy to 87.58%, leaving a gap of only 1.57 percentage points from RF. This demonstrates that the original large classical, deep difference was partly caused by unequal representation burden.

The complete confusion matrix identifies Ojet and Ov as the dominant directed confusion pair, with rates of 28.3% and 25.0%, respectively. Sensor analysis ranks $K_{c}$, $N_{pf}$, and $N_{po}$ as the three most informative measurements, and the top five sensors account for 47.62% of total RF importance. Cross-unit evaluation remains challenging: RF obtains a six-direction mean accuracy of 75.37%, whereas Transformer obtains 61.54%. Under five-shot training, KNN and RF achieve 71.24% and 70.82%, respectively, compared with 60.48% for 1D-CNN and 52.76% for LSTM. The sensor-subset ablation further shows that the Top-5 measurements achieve 87.62% diagnostic accuracy compared with 89.15% using all 19 sensors. This limited performance reduction indicates the potential for reduced sensor configurations, while the superior performance of the complete sensor set confirms that lower-ranked sensors still provide complementary diagnostic information.

These results support three engineering conclusions. First, input representation must be controlled before model families are ranked. Second, RF and KNN provide strong initial baselines for small, safety-critical datasets, while deep fusion models become competitive when domain-informed features are supplied explicitly. Third, cross-unit transfer, rather than within-unit accuracy alone, should be treated as a primary acceptance criterion for reusable-launch-vehicle health monitoring. Future work will extend the benchmark to larger multi-unit datasets, physics-guided fusion, domain adaptation, open-set diagnosis, and simulation-to-real validation.

## Figures and Tables

**Table 1:** Comparison of representative fault-diagnosis paradigms for liquid rocket engines.

Paradigm	Representative Techniques and Studies	Main Advantages	Main Limitations
Model-based methods	Physical modeling, residual analysis, parameter estimation, and extended Kalman filtering [3,4,5,6]	Strong physical interpretability; explicit relationships between component degradation and residual behavior; potential for fault localization	Require high-fidelity engine models and accurate parameters; sensitive to modeling uncertainty and unmodeled dynamics; limited portability across engine configurations
Signal-processing- based methods	PCA, FFT, dynamic time warping, parity-space analysis, and time–frequency analysis [7,8,9,10]	Do not require a complete physical model; can reveal fault-sensitive signal patterns; relatively low computational cost	Depend strongly on expert-designed features; sensitive to noise and operating-condition variation; features may not generalize across engines and fault severities
Conventional machine- learning methods	SVM, Random Forest, KNN, and conventional neural networks using hand-crafted or steady-state features [11,12,13,18,19,20]	Relatively high data efficiency; low training and inference cost; suitable for small-scale aerospace datasets	Performance depends on feature construction; limited capability to learn transient and long-term temporal behavior automatically
Deep-learning methods	CNN, LSTM, GRU, Transformer, and autoencoder-based diagnosis [14,15,16,17]	End-to-end nonlinear representation learning; capability to model temporal and cross- sensor relationships	Require more labeled samples; higher computational cost; sensitive to overfitting and distribution shift; limited interpretability
Generative and hybrid methods	GAN-based data augmentation and temporal generative models [10,23]	Can alleviate data scarcity and potentially combine diagnosis with synthetic-data generation	Training instability and mode collapse; physical consistency and diagnostic validity of generated samples require independent verification

**Table 2:** Composition of the XJTU-REF dataset used in this study.

Condition Code	Condition Type	Number of Samples
Ofu	Oxygen Secondary Line Clogging	114
Ojet	Oxidizer Injector Clogging	120
Op	Oxidizer-Pump-Efficiency Loss	120
Ov	Oxidizer Main Valve Fault	120
Rp	Fuel-Pump-Efficiency Loss	120
Rv	Fuel Main Valve Fault	120
Rxl	Gas Generator Fuel Leakage	147
ss	Combustion Chamber Ablation	120
Txl	Turbine Gas Leakage	125
H	Normal/Healthy Condition	3
**Subtotal: nine fault categories**		**1106**
**Total: fault and healthy conditions**		**1109**

**Table 3:** Representation-controlled performance on the nine-class fault-diagnosis task. Results are reported as mean ± standard deviation over the five stratified folds.

Method	Accuracy (%)	Macro-F1 (%)	Balanced Accuracy (%)	Training Time (s)	Common Input
RF	**89.15 ± 1.22**	**88.74 ± 1.31**	**88.80 ± 1.28**	0.163	19-D steady-state vector
KNN	88.31 ± 1.37	87.82 ± 1.45	87.91 ± 1.42	0.001	19-D steady-state vector
1D-CNN	86.42 ± 2.11	85.96 ± 2.24	86.03 ± 2.20	6.90	19 sensor tokens
LSTM	85.27 ± 2.46	84.71 ± 2.55	84.85 ± 2.51	21.80	19 sensor tokens
GRU	84.91 ± 2.57	84.33 ± 2.71	84.47 ± 2.64	18.60	19 sensor tokens
SVM	84.06 ± 1.48	83.42 ± 1.61	83.55 ± 1.55	0.010	19-D steady-state vector
Transformer	83.68 ± 3.01	82.94 ± 3.16	83.07 ± 3.09	24.40	19 sensor tokens

**Table 4:** Accuracy of deep models under three input-representation settings.

Model	Steady-State Only (%)	Sequence Only (%)	Sequence + Steady-State (%)	Fusion Gain (Points)
LSTM	85.27 ± 2.46	76.55 ± 3.72	86.83 ± 2.01	+10.28
GRU	84.91 ± 2.57	69.82 ± 4.11	85.96 ± 2.16	+16.14
1D-CNN	86.42 ± 2.11	64.02 ± 4.88	**87.58 ± 1.89**	+23.56
Transformer	83.68 ± 3.01	63.10 ± 5.20	85.21 ± 2.48	+22.11

**Table 5:** Per-class recall (%) under the representation-controlled setting.

Fault	SVM	RF	KNN	LSTM	GRU	1D-CNN	Transformer
Ofu	95.6	96.5	95.6	96.0	91.0	94.0	88.0
Ojet	74.2	56.7	68.3	72.0	75.0	78.0	76.0
Op	92.5	95.8	94.2	83.0	86.0	88.0	83.0
Ov	57.5	61.7	64.2	65.0	60.0	62.0	58.0
Rp	75.8	96.7	91.7	78.0	77.0	85.0	74.0
Rv	72.5	98.3	94.2	87.0	89.0	88.0	83.0
Rxl	98.0	96.6	98.0	98.0	97.0	97.0	96.0
ss	92.5	100.0	92.5	92.0	91.0	93.0	91.0
Txl	93.0	98.4	92.8	93.0	94.0	89.0	99.0

**Table 6:** Complete row-normalized RF confusion matrix (%). Rows are true classes and columns are predicted classes.

True/Pred.	Ofu	Ojet	Op	Ov	Rp	Rv	Rxl	ss	Txl
Ofu	**96.5**	0.0	0.0	1.7	0.0	1.8	0.0	0.0	0.0
Ojet	6.7	**56.7**	0.0	28.3	0.0	5.0	0.0	3.3	0.0
Op	0.0	0.0	**95.8**	0.0	4.2	0.0	0.0	0.0	0.0
Ov	4.1	25.0	0.0	**61.7**	2.5	6.7	0.0	0.0	0.0
Rp	0.0	0.0	3.3	0.0	**96.7**	0.0	0.0	0.0	0.0
Rv	0.0	0.0	0.0	1.7	0.0	**98.3**	0.0	0.0	0.0
Rxl	0.0	0.0	0.0	0.0	0.0	0.0	**96.6**	0.0	3.4
ss	0.0	0.0	0.0	0.0	0.0	0.0	0.0	**100.0**	0.0
Txl	0.0	0.0	0.0	0.0	0.0	0.0	1.6	0.0	**98.4**

**Table 7:** Largest directed confusion pairs extracted from the complete matrix.

True Fault	Predicted Fault	Confusion Rate (%)
Ojet	Ov	28.3
Ov	Ojet	25.0
Ojet	Ofu	6.7
Ov	Rv	6.7
Ojet	Rv	5.0
Op	Rp	4.2
Ov	Ofu	4.1
Rxl	Txl	3.4
Rp	Op	3.3
Ojet	ss	3.3

**Table 8:** RF sensor-importance ranking in the nine-class task.

Rank	Sensor	Category	Importance (%)
1	$K_{c}$	Combustion	12.09
2	$N_{pf}$	Pump	10.68
3	$N_{po}$	Pump	9.65
4	$Q_{o}$	Flow	7.98
5	$Q_{c}$	Flow	7.22
6	$RT_{t}$	Turbine	6.69
7	$Q_{f}$	Flow	6.64
8	$K_{p}$	Combustion	6.29
9	$RT_{c}$	Combustion	5.25
10	$RT_{p}$	Combustion	5.10

**Table 9:** Random Forest performance using different top-ranked sensor subsets.

Sensor Subset	No. of Sensors	Accuracy (%)	Macro-F1 (%)	Balanced Accuracy (%)
Top-3	3	82.93 ± 1.86	82.21 ± 1.94	82.35 ± 1.89
Top-5	5	87.62 ± 1.52	87.08 ± 1.61	87.17 ± 1.57
Top-8	8	88.54 ± 1.44	88.03 ± 1.52	88.11 ± 1.48
Top-10	10	88.91 ± 1.39	88.42 ± 1.48	88.50 ± 1.43
All-19	19	**89.15 ± 1.22**	**88.74 ± 1.31**	**88.80 ± 1.28**

**Table 10:** Mean cross-unit accuracy (%) over five repetitions for all seven methods. The final column is the difference between the within-unit controlled accuracy in Table 3 and the six-direction cross-unit mean.

Method	U1 → U2	U1 → U3	U2 → U1	U2 → U3	U3 → U1	U3 → U2	Mean	Mean Drop
RF	74.82	78.13	73.54	76.41	75.26	74.08	**75.37**	**13.78**
KNN	72.71	76.48	70.83	73.42	72.94	71.61	73.00	15.31
SVM	68.12	72.43	66.74	70.91	69.21	67.63	69.17	14.89
1D-CNN	68.24	71.63	65.12	69.37	67.81	66.32	68.08	18.34
LSTM	64.87	68.18	61.72	65.28	64.06	62.83	64.49	20.78
GRU	63.41	66.82	60.24	63.87	62.66	61.38	63.06	21.85
Transformer	61.82	65.37	58.94	62.58	60.73	59.81	61.54	22.14

**Table 11:** Few-shot accuracy (%) for all seven methods. The support-set size equals 9N because nine fault categories are evaluated. Results are mean ± standard deviation over five samplings.

*N*-shot	Support Size	SVM	RF	KNN	LSTM	GRU	1D-CNN	Transformer
1	9	40.80 ± 3.08	42.65 ± 3.35	**45.12 ± 3.21**	18.74 ± 4.12	17.26 ± 3.98	24.61 ± 4.05	15.83 ± 3.77
2	18	52.36 ± 3.01	55.91 ± 3.14	**57.43 ± 2.98**	31.52 ± 4.03	29.84 ± 4.16	38.92 ± 3.87	27.14 ± 4.24
3	27	58.91 ± 2.76	62.37 ± 2.85	**63.58 ± 2.69**	40.18 ± 3.92	38.66 ± 4.01	48.73 ± 3.61	35.61 ± 4.08
5	45	66.94 ± 2.38	70.82 ± 2.44	**71.24 ± 2.31**	52.76 ± 3.58	50.91 ± 3.71	60.48 ± 3.22	47.34 ± 3.86
10	90	70.23 ± 2.05	77.64 ± 2.11	**78.12 ± 1.98**	64.08 ± 3.14	62.47 ± 3.29	71.82 ± 2.77	59.76 ± 3.46
20	180	74.81 ± 1.84	**83.39 ± 1.76**	82.74 ± 1.81	73.46 ± 2.78	71.84 ± 2.91	79.37 ± 2.36	69.18 ± 3.08
Full	∼885	84.06 ± 1.48	**89.15 ± 1.22**	88.31 ± 1.37	85.27 ± 2.46	84.91 ± 2.57	86.42 ± 2.11	83.68 ± 3.01

## Data Availability

The original data presented in the study are openly available in https://github.com/gmy2013/A-Multi-Dimensional-Benchmark-of-Fault-Diagnosis-Methods-for-Liquid-Rocket-Engines (accessed on 3 September 2026).

## References

[1] National Aeronautics and Space Administration (2025). STS-51F. NASA Mission Archive.

[2] European Space Agency (2005). Ariane 5 ECA Qualification Flights.

[3] Maru Y., Mori H., Ogai T., Mizukoshi N., Takeuchi S., Yamamoto T., Yagishita T., Nonaka S. (2018). Anomaly detection configured as a combination of state observer and Mahalanobis-Taguchi method for a rocket engine. Trans. Jpn. Soc. Aeronaut. Space Sci. Aerosp. Technol. Jpn..

[4] Kanso S., Jha M.S., Galeotta M., Theilliol D. (2022). Remaining useful life prediction with uncertainty quantification of liquid propulsion rocket engine combustion chamber. IFAC-PapersOnLine.

[5] Lee K., Cha J., Ko S., Park S.Y., Jung E. (2018). Fault detection and diagnosis algorithms for an open-cycle liquid propellant rocket engine using the Kalman filter and fault factor methods. Acta Astronaut..

[6] Zeng Y., Shao X., Hu Q., Hu H., Pan H. (2021). Fault diagnosis of liquid rocket engine thrust chamber based on improved augmented particle filter. Proceedings of the 2021 40th Chinese Control Conference (CCC).

[7] Tsutsumi S., Hirabayashi M., Sato D., Kawatsu K., Sato M., Kimura T., Hashimoto T., Abe M. (2021). Data-driven fault detection in a reusable rocket engine using bivariate time-series analysis. Acta Astronaut..

[8] Bing L., Yulin Z. (1999). Parity space method for fault diagnosis of liquid rocket engine. J. Propuls. Technol. Beijing.

[9] Gao M., Renhf H. (2013). Independent component analysis for fault diagnosis of the liquid rocket engine. Missiles Space Veh..

[10] Deng L., Cheng Y., Shi Y. (2022). Fault detection and diagnosis for liquid rocket engines based on long short-term memory and generative adversarial networks. Aerospace.

[11] Ochella S., Shafiee M., Dinmohammadi F. (2022). Artificial intelligence in prognostics and health management of engineering systems. Eng. Appl. Artif. Intell..

[12] Gong Y., Chen Z. (2021). A sequential approach to feature selection in high-dimensional additive models. J. Stat. Plan. Inference.

[13] Zhu X., Cheng Y., Wu J., Hu R., Cui X. (2019). Steady-state process fault detection for liquid rocket engines based on convolutional auto-encoder and one-class support vector machine. IEEE Access.

[14] Li N., Xue W., Guo X., Xu L., Wu Y., Yao Y. (2019). Fault Detection in Liquid-Propellant Rocket Engines Based on Improved PSO-BP Neural Network. J. Softw..

[15] Xu L., Zhao S., Li N., Gao Q., Wang T., Xue W. (2019). Application of QGA-BP for fault detection of liquid rocket engines. IEEE Trans. Aerosp. Electron. Syst..

[16] Park S.Y., Ahn J. (2020). Deep neural network approach for fault detection and diagnosis during startup transient of liquid-propellant rocket engine. Acta Astronaut..

[17] Zhou Y., Chang B., Zou H., Sun L., Wang L., Du D. (2023). Online visual monitoring method for liquid rocket engine nozzle welding based on a multi-task deep learning model. J. Manuf. Syst..

[18] Li F., Chen J., Liu Z., Lv H., Wang J., Yuan J., Xiao W. (2022). A soft-target difference scaling network via relational knowledge distillation for fault detection of liquid rocket engine under multi-source trouble-free samples. Reliab. Eng. Syst. Saf..

[19] Lv H., Chen J., Wang J., Yuan J., Liu Z. (2021). A supervised framework for recognition of liquid rocket engine health state under steady-state process without fault samples. IEEE Trans. Instrum. Meas..

[20] Aiswarya N., Suja Priyadharsini S., Moni K. (2018). An efficient approach for the diagnosis of faults in turbo pump of liquid rocket engine by employing FFT and time-domain features. Aust. J. Mech. Eng..

[21] Xing D., Chen R., Qi L., Zhao J., Wang Y. (2020). Multi-source fault identification based on combined deep learning. MATEC Web Conf..

[22] Flora J.J., Auxillia D.J. (2020). Sensor failure management in liquid rocket engine using artificial neural network. J. Sci. Ind. Res..

[23] Cheng Y., Deng L. (2023). Application of Wasserstein distance in fault detection for liquid-propellant rocket engines. J. Natl. Univ. Def. Technol..

[24] Yuan L., Sun R., Zhai Z., Chen X. (2026). Interpretation of the XJTU-REF Liquid Rocket Engine Fault Simulation Dataset. Zhendong Gongcheng Xuebao/J. Vib. Eng..

